# Correction: Jarahian et al. Re-Expression of Poly/Oligo-Sialylated Adhesion Molecules on the Surface of Tumor Cells Disrupts Their Interaction with Immune-Effector Cells and Contributes to Pathophysiological Immune Escape. *Cancers* 2021, *13*, 5203

**DOI:** 10.3390/cancers17142362

**Published:** 2025-07-16

**Authors:** Mostafa Jarahian, Faroogh Marofi, Marwah Suliman Maashi, Mahnaz Ghaebi, Abdolrahman Khezri, Martin R. Berger

**Affiliations:** 1 German Cancer Research Center, Toxicology and Chemotherapy Unit Heidelberg, 69120 Heidelberg, Germany; martin.berger.hd@gmail.com; 2 Department of Hematology, Faculty of Medicine, Tabriz University of Medical Sciences, Tabriz 5165665931, Iran; marofif@tbzmed.ac.ir; 3 Stem Cells and Regenerative Medicine Unit at King Fahad Medical Research Centre, Jeddah 11211, Saudi Arabia; mmaashi@kau.edu.sa; 4 Cancer Gene Therapy Research Center (CGRC), Zanjan University of Medical Sciences, Zanjan 4513956184, Iran; sm.ghaebi@sina.zums.ac.ir; 5 Department of Biotechnology, Inland Norway University of Applied Sciences, 2418 Hamar, Norway; abdolrahman.khezri@inn.no

## Text Correction

There was an error in the original publication [1]. An explanation was missing regarding reference 20.

A correction has been made to Section “Introduction”, Paragraph 1:

Therefore, sialic acid has been repeatedly proposed as a possible target against tumor cells [11,20,24,25], ^a)^ of [20] see Supplementary Materials.

Supplementary Materials ^a)^: The study by Fossella et al. focuses on BB-10901, a humanized monoclonal antibody that selectively binds CD56. CD56 is an alternative name for NCAM, which is poly-sialylated. Multiple mechanisms underlie its antitumor activity. The connection to this article is reduction of electrostatic repulsion. BB-10901 binds to polysialylated CD56, potentially masking or neutralizing the negative charges of polySia. This can reduce electrostatic hindrance and allow closer physical contact between tumor and immune effector cells, promoting more effective immune recognition and lysis.

There was an error in the original publication. An explanation was missing regarding the selection of the literature. A correction has been made to Section “Materials and Methods”, Sub-section “2.2. Data Selection”, Paragraph 2:

The biological roles of heterophilic and homophilic membrane adhesion molecules in neuronal and embryonic development, and the development of certain neuronal diseases (such as Alzheimer’s disease, Parkinson’s disease, multiple sclerosis, and schizophrenia) ^b)^.

Supplementary Materials ^b)^: Re-expression of polysialylated CD56 (NCAM) is not only important for the immune escape of tumor cells but also central for the activation of Siglecs (e.g. CD33/Siglec-3 recognition and signaling). This suppressive signal is instrumental in neuronal diseases, eg. Alzheimer’s disease. In the current article, we focus on cancer, a subsequent publication will detail the role of CD56 polysialylation on various neuronal diseases caused by inflammation. However, we have selected the literature regarding neuronal diseases here to obtain an unbiased overview.

There was an error in the original publication. Reference 255 was misplaced. A correction has been made to Section “Results and Discussion”, Sub-section “3.8. Polysialylation of Glycoproteins Generates Diverse Functions”, Paragraph 4:

In fact, numerous publications have described that imbalances in sialic acid distribution and the degree of sialylated glycoproteins in conjunction with their co-partners are causal factors for certain neuronal diseases, such as Alzheimer’s disease [85,188,189,247,254,255], Parkinson’s disease [256], multiple sclerosis [257–259], and schizophrenia [260].

The authors state that the scientific conclusions are unaffected. This correction was approved by the Academic Editor. The original publication has also been updated.
