# Supplementary material for: Re-Expression of Poly/Oligo-Sialylated Adhesion Molecules on the Surface of Tumor Cells Disrupts Their Interaction with Immune-Effector Cells and Contributes to Pathophysiological Immune Escape
Source: Cancers (Basel). 2021 Oct 16;13(20):5203. doi: 10.3390/cancers13205203 (PMC8534074; doi:10.3390/cancers13205203)
Supplement: Supplementary file 1 [file cancers-13-05203-s001.zip › cancers-3764441-supplementary.pdf]

Supplementary Materials

File S1: **Information for PRIZMA search boxes**

Box1

((fibroblast growth factor receptor) OR FGFR OR (fibroblast growth factor) OR FGF OR (tyrosine-protein kinase Met) OR c-Met OR ROS OR (hepatocyte growth factor receptor) OR (HGFR) OR (hepatocyte growth factor) OR (ROS1 kinase) OR (ap-1 transcription factor subunit) OR (ROS1 gene fusions) OR (Proto-Oncogene 1) OR (NG2 chondroitin sulfate proteoglycan 4) OR (KDELR2 genes) OR (Endoplasmic Reticulum Protein Retention Receptor 2) OR (coiled-coil domain containing 6) OR (CCDC6 gene) OR (transmembrane protein genes) OR (actin binding protein genes) OR (actin binding proteins) OR ABP OR (anaplastic lymphoma kinase) OR ALK OR (Epidermal Growth Factor Receptor) OR EGFR OR (Epidermal Growth Factor) OR EGF OR (receptor tyrosine kinase) OR (tyrosine kinases) OR (Glial cell line-derived neurotrophic factor receptor) OR GDNFR OR (Glial cell line-derived neurotrophic factor) OR (GDNF) OR (Tropomyosin receptor kinase A) OR (Tropomyosin receptor kinase B) OR (TrkA/B) OR (Proto-oncogene tyrosine-protein kinase) OR (PSD95 and synaptophysin) OR (Tyrosinkinase Src) OR (Platelet-derived growth factor receptors) OR PDGF-R OR (GFRα1) OR (Platelet-derived growth factor) OR PDGF OR (complex formation with FGFR-1, ERK1 / 2, FAK and c-Met / ALK) OR (oncogenic pathways including PLCγ, STAT3, PI3K/AKT, VAV3, and MAPK/ERK) OR (Proto-oncogene tyrosine-protein kinase) OR (Leukocyte Receptor Tyrosine Kinase) OR (avian sarcoma RNA virus UR2) OR LTK OR
(Immunoglobulin Like Domains 3) OR LRIG3 OR (ROS1 fusion partner genes including Endoplasmic Reticulum Protein Retention Receptor 2) OR KDELR2 OR (ROS1 fusion partner genes including Coiled-Coil Domain Containing 6 (CCDC6) OR (ROS1 fusion partner genes including moesin gene) OR MSN OR (ROS1 fusion partner genes including transmembrane protein 106B gene) OR TMEM106B OR (ROS1 fusion partner genes including tumor protein D52 like 1 gene) OR TPD52L1 OR (ROS1 partner genes including tumor protein D52 like 1 gene actin binding 1 gene) OR LIMA1 OR (ROS1 fusion partner genes including clathrin heavy chain gene) OR CLTC OR (ROS1 fusion domain with binding domain of EGFR) OR TRKA OR (ROS1 signaling pathway components) OR (ROS1 partner genes in inflammatory myo-fibroblastic tumor) OR (ROS1 fusion partner including genes including CD74) OR (Solute Carrier Family 34 Member 2) OR SLC34A2 OR (syndecan 4 gene) OR SDC4 OR (ezrin genes) OR EZR OR (fused glioblastoma gene) OR FIG OR (ROS1 fusion partner genes including Tropomyosin 3) OR TPM3 OR (ROS1 fusion partner genes including Leucine Rich Repeats))
AND (polysialylate OR sialylation OR Neuroaminidases OR poly sialyltransferase OR sialic acids OR sialic acid) OR (NCAM 1) OR NCAM OR polySia OR (SynCAM 1) OR SynCAM OR (CADM 1) OR CADM OR (neuropilin 2) OR (NRP 2) OR NRP 2 OR (neuropilin 1) OR neuropilins OR (NRP 1))
AND (cholangiocarcinoma OR ovarian cancer OR gastric cancer OR colorectal cancer OR angiosarcoma OR (spitzoid melanoma) OR (non small cell lung cancer) OR NSCLC OR angiogenesis OR tumorigenesis OR apoptosis OR Proliferation OR Plasticity OR (Cell Migration))

6165 articles, 260 duplicates, 130 hits, after removal of duplicate hits: 82 hits

Box2

(Integrins OR CD43 OR CD45 OR CD29 OR CD7 OR CD44 OR CD49b OR VLA-2 OR CD44v6 OR CD11b OR CD18 OR (E selectin) OR (L selectin) OR (P selectin) OR Galectins OR Galectin 1 OR Galectin 3 OR Galectin 9 OR (homodimeric galectin) OR plexin OR SEMA3F OR CDCP1 OR TSP-1 OR (pro-inflammatory immune checkpoint receptors) OR (anti-inflammatory check point receptors) OR PD-1 OR PD-L1 OR B7-H1 OR CD95 OR CTLA-4 OR (T cell hyperactivity) OR (T-cell differentiation) OR (S-type lectins) OR (galectin-1- induced cell death) or (galectin-9 -induced cell death) OR (cell adhesion glycoproteins) OR EGFR OR TRAILR OR TNFR OR PECAM) AND ((polysialic acid) OR (alpha-2,8 sialic acid) OR (alpha-2,8 sialic acid) OR ST8Sia OR polysialylation OR polysialyltransferase OR repulsion OR sialyltransferases OR (N-Acetylneuraminic Acid) OR (Sialic Acids/biosynthesis) OR (N-glycolylneuraminic acid) OR Neu5Gc OR KDN OR (S-type lectins) OR (fluorinated sialic acid) OR (NRP-2 / EGFR-complex) OR (unsialylated growth factor receptors) OR (N-glycosylation of CD25) OR ST6Gal I OR (N- glycans) OR (O-glycans))
AND (Plasticity OR (Cell Migration) OR Metastasis OR apoptosis OR (homophilic interaction) OR (heterophilic interaction) OR (T-cell differentiation) OR (TCR signal transduction))

4223 articles, 168 duplicates, 84 hits, after removal of duplicate hits: 42 hits

Box3

(((Polysialic acid OR sialic acids OR Sialic acid OR Polysialic acids OR N-glycosylation OR O-glycosylation OR sialyltransferases OR (glycosyltransferases/metabolism) OR (N-Acetylneuraminic Acid) OR (Neu5Gc) OR (KDN ligand) OR (KDN) OR (Neu5Gc) (sialoglycoconjugates to modify lectin-ligand interaction) OR CD57 OR repulsion OR (sialoglycoconjugates to modify lectin-ligand interaction) OR (Lymphoproliferative Disorders/complications)) AND (tumor OR tumour OR cancer OR (cell death) OR (Dead domains) OR (resistant tumor cells) OR Plasticity OR Cell Migration OR (homophilic interaction) OR heterophilic interaction) OR cell adhesion glycoproteins OR ((Apoptosis-Inducing Ligand OR Apoptosis OR phagocytosis OR autophagy (Apoptosis/drug effect) OR TNF-Related OR Fas-ligand OR stem cells OR CD95 OR EGFR OR TRAILR OR (TRAILR trafficking) OR TNFR OR (EGF repeat) OR (Notch signaling)) AND (inducing apoptosis) OR Apoptosis Regulatory OR apoptotic OR Metastasis OR immune escape OR embryogenesis OR Angiogenesis OR (cluster formation) OR (cluster formation of glycoproteins) AND ((Lectins OR lectin OR (S-type lectins) OR galectins OR Galectin OR (Galectin 1) OR (Galectin 3) OR (Galectin 8) OR (Galectin 9) OR (homodimeric galectins) OR ((selectins OR selectin OR CD62 e/p/l OR e selectin OR p-selectin OR l-selectin OR CD31 OR CD34 OR Homing OR CD44 OR plexin A/B OR hyaluronate OR (Hyaluronan Receptor) OR leukosialin OR T-Cells)))
64987 articles, 128 duplicates, 64 hits, after removal of duplicate hits: 26 hits
Box 4
((fibroblast growth factor receptors) OR (fibroblast growth factor receptor) OR (tyrosine-protein kinase Met) OR (receptor tyrosine kinase) OR (ROS1) OR ALK OR EGFR OR (ezrin gene) OR (Proto-Oncogene 1) OR (fusion partner genes) OR (syndecan 4 gene) OR (KDELR2 gene) OR (coiled-coil domain containing 6) OR (transmembrane protein genes) OR (actin binding protein gene) OR (Glial cell line-derived neurotrophic factor) OR (neurotrophic factor) OR (Tropomyosin receptor kinase A) OR (Proto-oncogene tyrosine-protein kinase) OR (Epidermal Growth Factor Receptor) OR (Leukocyte Receptor Tyrosine Kinase) OR NCAM1 OR CADM1 OR neuropilin OR NRP 2 OR NRP 1 OR (SynCAM 1) OR Integrins OR CD45 OR CD29 OR CD44 OR CD49b OR CD49b OR CD44v6 OR CD49b OR VLA-2 OR CD44v6 OR CD11b OR CD18 OR CD18) OR Galectins OR galectin OR (Galectin 1) OR (Galectin 3) OR (Galectin 9) OR (Galectin 7) OR (Galectin 10) OR (homodimeric galectin) OR (Galectin cluster) OR (Galectins (S-type lectins) induce apoptosis))
AND (polysialylate OR sialylation OR Neuroaminidase OR (poly sialyltransferase) OR (sialic acids) OR (sialic acid) OR polySia OR autophagy OR (cluster formation of glycoproteins))
AND (Tumour OR Tumor OR OR apoptosis OR Proliferation OR Plasticity OR (Cell Migration) OR repulsion)
7276 articles, 86 duplicates, 43 hits, after removal of duplicate hits: 14 hits
Box 5
((Galectins) OR (Galectin 1) OR (Galectin 2) OR (Galectin 3) OR (Galectin 4) OR (galectin 5) OR (Galectin 6) OR (Galectin 7) OR (Galectin 8) OR (Galectin 9) OR (Galectin 10) OR (homodimeric galectins) OR (S-type lectins) OR autophagy OR (glycoprotein cluster formation) OR (galectins mediated T-cell differentiation) OR (fluorinated sialic acid) OR (galectin-1/galectin-9 induced cell death) OR (CD25 N-glycosylation) OR ST3Gal OR ST6Gal OR (N- OR O-glycans) OR (TSP-1) OR (Galectin-9/Galectin-1 induced T cell apoptosis Pathways) OR (Antigens promote tumor resistance) OR (Sialic acid Blockade suppresses tumor growth) OR (Siglecs/Selectins/Galectins create Turning-Off Signalling) OR EGFR OR HGFR OR (hepatocyte growth factor) OR (Galectin cluster) OR (Galectin induced apoptosis) OR (autoimmunity via glycosylated galectin 1) OR (Galectin-9/Galectin-1 induced T cell apoptosis Pathways) OR (Siglecs, Selectins, Galectins Turn-Off Signals) OR (ST6Gal-I regulates macrophage apoptosis) OR (tumour galectin-1 mediated T-cell apoptosis) OR (galectin mediated effector self/non-self-recognition) OR (E selectin) OR (L selectin) OR (P selectin) OR (Fucosylated CD44) OR (Galectin-1 mediated effector regulation T-CD4+CD25+ cells) OR (tumour immune evasion) OR angiogenesis OR tumorigenesis OR apoptosis OR metastasis OR (immune escape) OR (inhibitory receptors) OR (resistant tumor cells) OR TRAILR OR TNFR OR (Death domain Receptor) OR (Apoptosis/drug effects) OR (Apoptosis Regulatory) OR (TNF-Related Apoptosis-Inducing Ligand) OR (TRAIL trafficking) OR (Immunoglobulin mucin3) OR (TIM3) OR (Mucins) OR (blockaded TIM3 negative immune checkpoint) OR (tyrosine kinase receptor)) AND ((polysialic acid) OR (sialic acids) OR (Sialic acid) OR sialyltransferases OR (alpha-2,8 sialic acid) OR (alpha-2,6 sialic acid) OR (Sialyl LewisX) OR (sialylated Glycans) OR (targeted therapies in non-small cell lung cancer OR EGF) OR (transcriptional landscape

OR mutational profile of lung adenocarcinoma) OR (Epidermal Growth Factor Receptor Cell Proliferation Signaling Pathways) OR (chimeric receptors of epidermal growth factor receptor or c-Ros))
50516 articles, 300 duplicates, 150 hits, after removal of duplicate hits: 132 hits
Box 6
(overexpression OR hypersialylation OR (sialic acid in resistant tumor cells) OR (sialic acid in stem cells) OR (sialic acid and chemotherapy treatment) OR (polysialylated glycans) OR polysialylation OR sialylation OR (N-acetyl neuraminic acid) OR polysialyltransferases OR polysialyltransferase OR (sialic acid) OR (sialic acids) OR polysia OR repulsion OR homophilic interaction) AND (neural cell adhesion molecule) OR NCAM OR L1-CAM OR CD56 OR (CADM 1) OR CADMs OR (SynCAM 1) OR neuropilin OR neuropilins OR N-glycan OR HNK1 OR CD57 OR N-Cadherin OR E-cadherin OR (SynCAM 2) OR L1CAM OR (Neuropilin 2) OR (neuropilin 1) OR (Heparan sulfate) OR proteoglycan OR CD44 OR CD44v6 OR NRP2 OR NRP1 OR (EGFR 2) OR c-Met OR Plexin A/B OR c-Met OR (Plexin A/B) AND cancer OR tumor re-expressed polysialylated adhesion molecules NCAM1 NRP2 OR Syn-CAM AND (OR metastasis OR apoptosis OR migration OR proliferation OR (adaptive immune system) OR (sialic acid inducing CD27 on lymphocytes) OR (sialic acid inducing CD70 in tumors) OR RAS gene OR innate immune system)
44098 articles, 150 duplicates, 75 hits, after removal of duplicate hits: 15 hits
Box 7
((NCAM 1) OR (SynCAM 1) OR (CADM 1) OR (NRP 2) OR (NRP 1) OR (neuropilin 2) OR (neuropilin 1) OR neuropilins OR repulsion)) AND ((polysialylated glycans) OR (poly sialylation) OR polySia OR sialylation OR (n acetylneuraminic acid) OR (poly sialyltransferases) OR (poly sialyltransferase) OR (sialic acids) OR (sialic acid) AND (Overexpression OR hypersialylation OR (upregulation of sialic acid inducing CD70 in tumors) OR (inducing CD27 on lymphocyte) OR (upregulation of sialic acid resistance in tumor cells) OR (upregulation of sialic acid in stem cells) OR (upregulation of sialic acid in drug resistant tumor cells) OR (sialic acid))
17062 articles, 174 duplicates, 87 hits, after removal of duplicate hits: 5 hits
Box 8
(sialic acid binding receptors OR "sialic acid binding immunoglobulin like lectins" OR sialylated bacteria OR viruses envelope OR sialylated pathogens OR "sialic acid binding ig like lectin 1" OR mannose 6 phosphate OR "sialic acid binding ig like lectin 2" OR C type lectins OR lectins on epithelial cells OR lectins on lymphocyte cells OR lectins on platelet cells OR Lectins in plants OR Lectins in animals OR Lectin in viruses OR Lectin on bacteria OR virus hemagglutinin OR Selectins on epithelial cells (homing process of immune-system cells) AND (I type lectins) OR siglecs OR lectins OR siglec 1 OR (Sigle 1 CRD) OR sialoadhesin OR CD22 OR siglec 2 OR siglec 3 OR CD33 OR siglec 5 OR siglec 6 OR siglec 7 OR siglec 8 OR siglec 9 OR siglec 10 OR DAP-12 OR ITIM OR IL10 OR "DNAX-activating protein" OR Siglec 3 interact with NCAM 1) OR siglec 4 OR MAG OR "myelin associated glycoprotein" OR Siglecs OR "natural killer cells" OR siglec-15 OR siglec-15 conserved in mammals OR alzheimer'sdisease OR multiple sclerosis OR (multiple sclerosis disease OR MS OR anti NCAM OR "multiple sclerosis disease" OR Parkinson’s disease OR Schizophrenia’s disease OR β-Amyloid OR beta-Amyloid OR β-Amyloid recycling OR Glia cells) AND (sialic acids OR immune escape OR humoral OR adaptive innate immune system OR effector cells)
24210 articles, 138 duplicates, 69 hits, after removal of duplicate hits: 11 hits
Box 9
((neural cell adhesion molecule) OR NCAM OR NCAM1 OR NCAM140 OR NCAM180 OR NCAM120 OR PSA) OR ((homophilic interaction) OR (heterophilic interaction) OR SynCAM1 OR (CADM 1) OR TSCL1 OR CD36 OR (SynCAM 2))
OR ((neuropilin 2) OR (neuropilin 1) OR NCAM2 OR OR NCAM2/OCAM/RNCAM OR lactoferrin OR polysialylated OR sialylation OR (poly sialylation) OR polySia OR Neu 1 OR Neu 2 OR Neu 4 OR Neu 3 OR sialyltransferase OR poly sialyltransferase OR n-acetylneuraminic acid OR (sialic acids))

96944 articles, 606 duplicates, 303 hits, after removal of duplicate hits: 81 hits

Box 10

(polysialic acid OR alpha-2,8 sialic acid OR sialyltransferases OR ST8Sia OR poly sialylation OR repulsion OR polysialyltransferase OR N-Acetylneuraminic Acid OR Sialic Acids biosynthesis OR Sialyl LewisX OR Lewis X Antigen OR Sialyl Lewis X Antigen OR ST3GAL4 Expression OR sialylated Glycan OR Sweet angiogenesis OR synaptic plasticity OR SynCAM 1 target for polysialylation) AND (FGFR2 Amplifikation) OR (fibroblast growth factor receptors) OR (EGFR 2) OR (FGF-2-mediated FGFR1 signal) OR (Epidermal growth Factor Receptor) OR (EGFR) OR (vascular endothelial Growth Factor receptor) OR (Characterization novel vascular endothelial growth factor receptors) OR (VEGFR2) OR (VEGFR3) OR (VEGFR1) OR (vascular endothelial Growth Factor) OR (VEGFR structure-function analysis) OR (VEGFR2 internalisation via micropinocytosis) OR (C-Met) OR (Plexin A/B) OR (CD44) OR (synaptophysin) OR (neuronal/vascular/endothelial Growth Factor) OR (Epidermal Growth Factor) OR (EGF) OR (neuron-glial antigen 2 myelinisation) OR (NG2; chondroitin sulfate proteoglycan 4) OR (HNK1) OR (E-cadherin) OR (N-cadherin) OR (Cadherins) OR (mice transgenic Rip1Tag2 NCAM) OR (carcinogenesis model pancreatic β-cell) OR (tumor metastases) OR (upregulating lymph angiogenesis) OR (hepatocyte growth factor receptor) OR (HGFR) OR (hepatocyte growth factor) OR (transcription factor ap-1 subunit) OR (Angiogenesis) OR (tumorgenesis) OR (Endoplasmic Reticulum Protein Retention Receptor 2) OR (Glial cell line-derived neurotrophic factor) OR (GDNFR) OR (GDNF) OR (synaptophysin PSD95) OR (Platelet-derived growth factor receptors) OR (PDGFR) OR (Platelet-derived growth factor) OR (PDGF) OR (Cell Differentiation) OR (ErbB-2 Receptor) OR (rapamycin signal axis) OR (glioblastoma) OR (Tumor Suppressor Protein p14ARF) OR (Lymphoma, T-Cell) OR (Novel RUNX1 isoforms) OR (acute myeloid leukemia) OR (Annexin A5) OR (NF-kappa B nfk antagonist) OR (ST3GAL4 Expression) OR (c-Met activation) OR (ICAM-1) OR (PI3K/AKT pathway) OR (MART-1 Antigen) OR (PTEN Phosphohydrolase) OR (c-akt) OR (Hyaluronan Receptors) OR (Hyaluronic Acid) OR (metabolism) OR (Human Diseases Nutritional Antioxidants) OR (neurodegenerative diseases) OR (oxidative stress) OR (Tumor biomarkers) OR (Pancreatic Neoplasms) OR (Claudin-5) OR (Akt/CREB) OR (Bdnf) OR (Bcl-2) OR (Nobiletin) OR (Gene Rearrangement) OR (Etv6) OR (Ntrk3) OR (Neuropilin-1/GIPC1 signaling) OR (TRKA) OR (carrier Proteins) OR (Phosphotransferases) OR (derived growth factor-BB-induced fibroblast migration via hyaluronan receptor CD44) OR (hyaluronan activation) OR (astrocyte-induced synaptogenesis) OR (Neurogenesis) OR (microenvironment Exploiting NK Cell Surveillance Pathways) OR (immunotherapy) OR (VEGF induces angiogenesis signalling) OR (Vascular Endothelial Growth Factor A) OR (Membrane trafficking) OR (synaptic adhesion molecule) OR (drives synapse assembly) OR (low malignant potential) OR (low grade ovarian serous tumours) OR (Cancer Fucosylated Antigens) OR (fucosyltransferases) OR fucosylation) OR complement factor H is relevance disease) OR Arthritis, Rheumatoid immunology) OR Metabolic sialic acid blockade activation moDCs) OR Toll-Like receptors) OR Sweet angiogenesis) OR CD4-Positive T-Lymphocytes immunology) OR CD8-Positive T-Lymphocytes immunology) OR T-cell acute lymphoblastic leukemia expressed NCAM) OR small cell carcinoma expressed NCAM) OR Poly sialylated NCAM1 Regulate Brain Development) OR SynCAM1 poly sialylation as target) OR CNS synaptogenesis molecular mechanisms) OR NCAM isoforms specific detection) OR proteoglycan Glypican-1 binds VEGF165) OR (factors semaphorins bind NPR2/NPR1/NCAM) OR Neuropilin-2 expression promotes epithelial/mesenchymal transition) OR (metastatic melanoma regulation via ICAM-1 critical role tumors) OR (Adult neurogenesis) OR (psychiatric diseases via brain injury role) OR (Structural features close homologue L1-CAM/CHL1) OR (Poly sialic acid blocks mononuclear phagocyte reactivity) OR (microglia) OR (Functional interaction VEGF-C/VEGF-D/neuropilin receptors) OR (T-cell infiltration regulating mechanisms) OR (SEMA3F/VEGFR/NP1/NP2 Interaction) OR (NCAM2-mediated synaptic adhesion) OR (novel mechanism underlying malformations resulting) OR (ST6Gal-I regulates macrophage apoptosis via alpha2-6 sialylation) OR (Cadherin signaling mediated cell-cell adhesion) OR (FGF mutations promote mammalian higher glycosylation) OR (Glycoengineering therapeutic potential) OR (Autoimmunity) OR (siglecs) OR (Anti-CD137 antibodies) OR (tumors CD56 expression clinical significance) OR (CD44 isoforms via ErbB ligands) OR (small cell lung cancer targeted molecules) OR (NCAM2 Regulates axonal Dendritic) OR (MAP2) OR (immunotherapy Glycosylation as checkpoint) OR (SynCAM 1 Polysialylation via polys sialyltransferase ST8SiaII) OR (nervous system plasticity development via NCAM) OR (CNS Polysialic Acid) OR (BDNF/TRKB and BDNF/p75NTR signaling networksystem) OR (SynCAM/neuroligin) OR (Polysialylated NCAM represses E-cadherin) OR (vascular morphogenesis by inhibiting integrin function) OR (Th1 polarization via Sialic acid removal) OR (protein stability via glycosylation pharmaceuticals Effects) OR (Autophagy control via VEGF-C/NRP2) OR (Myeloid leukemias express VEGF receptors including neuropilins) OR (Glycans biological roles) OR (mimic cell-cell junctions Lectin-mediated protocell crosslinking to via Lectin mediated crosslinking) OR (E-

Cadherin suppress Invasion) OR (L1CAM expressing levels correlated glioblastoma/metastatic brain tumors) OR (cell Proliferation Signaling Pathways) OR (stem cell-derived oligodendrocyte precursor cells) OR (transforming growth factor-beta induced Neuropilin-2) OR (neural stem cells glycobiology) OR (hyaluronan receptor RHAMM regulates immune escape) OR (TGF-beta-induced Foxp3+ T cells) OR (Proteolytic processing converts repelling signal Sema3E) OR (NCAM-140/NCAM-120) OR (NCAM and FGFR1 co-expression) OR (blood vessel growth Molecular mechanisms) OR (Insulin/IGF-I signaling pathways enhances tumor cell invasion) OR (L1-CAM binds ErbB receptors) OR (Phalloidine) OR (anti-VEGF therapy) OR (cancer Glycosylation) OR (tumor immune evasion) OR (Angiogenesis) OR (tumorgenesis) OR (Cell Proliferation) OR (Signaling Pathways Plasticity) OR (tumor metastases) OR (Cell Migration) OR (Metastasis) OR (apoptosis) OR (cell adhesion glycoproteins) OR (proliferation) OR (migration) OR (carcinogenesis) OR (upregulating lymphangiogenesis) OR (Sweet glycosylation) OR (Cell Proliferation Signaling Pathways) AND (cell adhesion glycoproteins) OR (NCAM 1) OR (NCAM 140) OR (NCAM 180) OR (NCAM 120) OR (L1-CAM) OR (CD56) OR (SynCAM) OR (SynCAM 1) OR (CADM1) OR (CADM) OR (Neuropilin-2) OR (NPR2) OR (NPR1) OR (neuropilin 1) OR (neural cell adhesion molecule)
107241 articles, 514 duplicates, 257 hits, after removal of duplicate hits: 26 hits
Box 11
(((CD45 OR CD29 OR CD44 OR CD49b OR VLA-2 OR CD44v6 OR CD11b OR CD18 OR E selectin OR L selectin OR OR P selectin OR Galectins OR Galectin 1 OR Galectin 3 OR Galectin 9 OR (homodimeric galectin) OR plexin OR SEMA3F OR CDCP1 OR TSP-1 OR (pro-inflammatory immune checkpoint receptors) OR (anti-inflammatory check point receptors) OR PD-1 OR PD-L1 OR B7-H1 OR CTLA-4 OR (T cell hyperactivity) OR (S-type lectins)) AND (poly sialylation OR Sialylation polysialyltransferase OR sialyltransferase OR ST8SiaIV OR ST8SiaII OR ST3SiaII OR ST6Sia-I OR N-Acetylneuraminic acid OR sialic acid OR sialic acids OR check point OR differentiation)))
27900 articles, 92 duplicates, 46 hits, after removal of duplicate hits: 9 hits
Box 12
(overexpression OR hypersialylation OR (sialic acid inducing CD70 in tumors[MeSH Terms] OR (sialic acid inducing CD27 on lymphocytes[MeSH Terms] OR (sialic acid in resistant tumor cells[MeSH Terms] OR (sialic acid in stem cells[MeSH Terms] OR (sialic acid in drug-resistant tumor cells[MeSH Terms] OR (sialic acid and chemotherapy treatment[MeSH Terms] OR (polysialylated glycans[MeSH Terms] OR polysialylation OR sialylation OR (N-acetyl neuraminic acid[MeSH Terms] OR polysialyltransferases OR polysialyltransferase OR (sialic acid) OR (sialic acids) OR polysia OR repulsion OR homophilic interaction[MeSH Terms] OR heterophilic interaction[MeSH Terms] tyrosin kinase receptors OR Proliferation OR angiogenesis OR metastasis OR immune escape OR cell-cell adhesion OR Natural killer cells OR T-CD8 OR NKT cells OR MHC-I OR Inhibitory receptors OR activating receptors AND (neural cell adhesion molecule[MeSH Terms] OR NCAM OR L1-CAM OR CD56 OR (CADM 1) OR CADMs OR (SynCAM 1) OR neuropilin OR neuropilins OR N-glycan OR HNK1 OR E-cadherin OR SynCAM 2 OR L1CAM OR Neuropilin 2 OR NRP2 OR NRP1 OR VEGFR OR EGFR 2 OR c-Met OR Plexin A/B OR c-Met OR Plexin A/B)
80743 articles, 300 duplicates, 150 hits, after removal of duplicate hits: 9 hits
Box 13
(metastasis OR angiogenesis OR resistant tumor cells OR migration OR apoptosis OR (Ras kinases) OR (tyrosin kinase) OR proliferation OR (Immune escape) OR (T cell differentiation) OR (innate immune natural killer cells[MeSH Terms]) OR (adaptive immune killer cells[MeSH Terms]))
AND (lactoferrin OR transferrin OR (iron canals) OR (Human Milk has Polysialic acid) OR antibacterial OR antiviral OR (IgA protease) OR (Human proteolytic activity) OR (Oral Lactoferrin) OR (CD57 antigen) OR CD44 OR (polysialylated NCAM reexpressed via different tumors[MeSH Terms]) OR (signal transduction) OR Siglecs OR siglec3 OR siglec1 OR Integrins OR (alpha integrin) OR (beta integrin) OR galectins OR (Gal 1) OR (Gal 5) OR (Gal 9) OR selectins OR (Fas-Ligand) OR CD95 OR hypoxia OR (transcription factor HIF-1) OR (oxidative stress) OR E-cadherin OR L1-CAM OR ICAM OR Claudins OR homing OR (tissue lymphocyte infiltrating) OR inflammatory OR (Effector cells) OR (autoimmune disease) OR (beta amyloid) OR (TNF-alpha) OR (TGF-beta) OR (Heparan sulphate) OR (transcription factor HIF-1[MeSH Terms]) OR hypoxic OR (Hypoxia conditions) OR (environmental factors) OR (angiotensin II) OR pyruvate OR (Glucosamine-N-acetate) OR lactate OR (mitochondrial pyruvate carrier[MeSH Terms]) OR hypoxic OR (phosphophenol-pyruvate-derived moiety[MeSH Terms]) OR Aldolase) OR (NCAM OR NCAM1 OR NCAM140 OR NCAM2 OR NCAM-180 OR NCAM120 OR psa-ncam OR (homophilic

interaction) OR (heterophilic interaction) OR SynCAM1 OR SynCAM2 OR CADM1 OR CADM2 OR TSCL1 OR neuropilin2 OR neuropilin-2 OR NRP-2 OR NRP-1 OR neuropilin1 OR neuropilins OR (Growth factor receptors) OR (neuronal disease) OR (Parkinson disease) OR (Multiple sclerosis disease) OR (schizophrenia disease) OR (Alzheimer's disease) OR sphingomyelin OR MAG OR (ganglioside GM1/GM2/GM3/GD1a/GT1b) OR anti-NCAM OR CD33 OR siglec3 OR Siglec4)
AND (polysialylated OR polysialylated OR sialylation OR (poly sialylation) OR (neural cell adhesion molecules) OR (a-2,8-sialyltransferase) OR (sialic acids) OR (Polysialic acid) OR (Sialic acid) OR sialyltransferases OR (sialic acids poly sialyltransferases)
34244 articles, 490 duplicates, 245 hits, after removal of duplicate hits: 4 hits
Box 14
(factor semaphorin classe3 plexin A) OR (innate immune system/Alzheimer's disease) OR (network signaling system via BDNF/ TRKR, BDNF/p75NTR OR class-3 semaphorins/neuropilin receptor promote autoimmunity dependent galectin1) OR (BDNF/TrkB signaling regulates HNK-1 carbohydrate expression promotes functional recovery after peripheral nerve repair) OR (Chromosome 3 anomalies investigated via genome wide SNP analysis, low malignant potential, low grade ovarian serous tumours) OR (astrocyte Molecular mechanisms induced synaptogenesis/neurogenesis) OR (Pancreatic cancer cell glycosylation regulates cell adhesion and invasion via alpha2beta1 integrin, E-cadherin function modulation) OR (Variant glycosylation lead beta1 integrins regulatory mechanism) OR (NCAM/FGFR signalling complex disseminated tumor metastatic) OR (breast cancer-associated carbohydrate antigens) OR (glioblastoma FIG/ receptor tyrosine kinase ROS Fusion interstitial) OR (ROS fusion tyrosine kinase activates SH2 domain-containing phosphatase-2/phosphatidylinositol 3-kinase/mammalian target via mice glioblastoma rapamycin signalling axis) OR (TGF-beta-induced Foxp3+ T cells via controls IL-expression stability) OR (anaplastic lymphoma kinase/ cancer pathogenesis) OR (Proteolytic processing converts Sema3E repelling signal inducer lung metastasis) OR (invasive growth) OR (blood vessel growth Molecular mechanisms OR CD33(+)/p-STAT1(+) double-positive cell gastric cancer prognostic factor) OR (anti-VEGF-therapy) OR (solid tumor malignancies Angiogenesis inhibition) OR (detection of ALK, ROS1, RET, NTRK1 fusions in non-small cell lung carcinoma via Clinical implementation anchored multiplex PCR) OR (mouse brain CD9 is associated with α6/β1 integrin and neural adhesion molecule L1 which induced neurite outgrowth, cell migration invitro) OR (Polysialic Acid in the CNS promote Plasticity) OR (Glycosylation in cancer mechanisms and clinical implications) OR (An antimicrobial protein, lactoferrin find via sweat proteomic analysis) OR (Adult neurogenesis and its brain and psychiatric diseases role) OR (tyrosine kinase signalling Survey reveals human ROS kinase fusions in cholangiocarcinoma) OR (colorectal cancer cells expressed Neuropilin-2, which promotes TGF-beta1-mediated epithelial to mesenchymal transition) OR (Membranes Polyether sulfone Prepared with 3-Aminopropyltriethoxysilane Modified Alumina Nanoparticles that Removed Cu2+ from Water) OR (Breakpoint analysis of the transcription genome profiles reveals new gene fusions that span multiple human cancers) OR (interfibrillar proteoglycan structure bridges shape modules in extracellular matrix OR (Shaping Synapses via Neural Extracellular Matrix) OR (CNS synaptogenesis Molecular mechanisms)
101422 articles, 100 duplicates, 50 hits, after removal of duplicate hits: 25 hits
Box 15
(Lactoferrin OR transferrin OR Hypoxia HIF-1 OR aldolase OR lactates) AND (Tumor OR Polysialic acid)
71798 articles, 330 duplicates, 165 hits, after removal of duplicate hits: 9 hits
Box 16
((angiogenesis OR tumorigenesis OR apoptosis OR TRAIL OR TNFR OR Dead domain rezeptor OR immune escape OR resistant tumor cells[MeSH Terms] OR metastasis) AND ((galectins OR galectins OR Galectin-1 OR Galectin-2 OR Galectin-3 OR Galectin-7 OR Galectin-8 OR Galectin-9 OR Galectin-10 OR (homodimeric galectin[MeSH Terms] OR autophagy[mesh] OR (glycoprotein cluster formation[MeSH Terms] OR (galectins mediated T-cell differentiation[MeSH Terms] OR (Galectin-3 fluorinated sialic acid[MeSH Terms] OR galectin-1 OR (galectin-9 induced cell death[MeSH Terms] OR (N-glycosylation CD25[MeSH Terms] OR ST3Gal OR ST6Gal OR N-glycans and O-glycans OR TSP-1[mesh] OR (Sialic acid blocked Growth factor receptor[MeSH Terms] OR (hepatocyte growth

factor[MeSH Terms] OR (Galectins induced apoptosis[MeSH Terms] OR Galectins cluster OR (S-type lectins[MeSH Terms] OR (autoimmunity via glycosylated galectin-1[MeSH Terms] OR (Galectin-9/Galectin-1 induced T cell apoptosis signal[MeSH Terms] OR (TNF-Related Apoptosis-Inducing Ligand[MeSH Terms] OR (Siglecs/Selectins/Galectins Turn-Off Signal[MeSH Terms] OR (ST6Gal-I regulates macrophage apoptosis [MeSH Terms] OR (tumor galectin-1 mediated T-cell apoptosis[MeSH Terms] OR (galectins mediated effector self/non-self-recognition[MeSH Terms] AND (polysialic acid OR sialic acids OR sialyltransferases OR Neuraminidase OR Neu 1 OR Neu 2 OR Neu 3 OR Neu 4 OR 2,6 sialic acid OR sialidase))

59567 articles, 120 duplicates, 60 hits, after removal of duplicate hits: 42 hits

Box 17

((immune escape OR resistant tumors OR apoptosis OR resistance pathogens OR Bacteria or Virus OR proliferation OR Inhibition) AND ((Phlebo virus's receptor DC-SIGN induced Endocytosis[MeSH Terms] OR adenovirus 52 Polysialic acid receptor's adenovirus 52 induced cellular growth factor BB[MeSH Terms] OR Becaplermin receptor CD44 [MeSH Terms] OR hyaluronan receptors RHAMM /CD44[MeSH Terms] OR complement factor H[MeSH Terms] OR Essential carbohydrates neuronal-development are sialic acids[MeSH Terms] OR capsular Streptococcus expressed sialic acid, which blocked phagocytosis and promote resistance tumor cells[MeSH Terms] OR dendritic cells transmembrane DC-SIGN binds virus particle proteins[MeSH Terms] OR DC-SIGNR/DC-SIGN bind Ebola virus/HIV/HCMV/NDV/vaccinia virus/hepatitis C viruse/Lassa virus/influenzas[MeSH Terms] OR bacteria's mimic Sialic acid so Modulates Distinct Histones antimicrobial ability[MeSH Terms] OR dendritic cells Sialic acid removal improves antigen cross-presentation[MeSH Terms] OR many tumors re-expressed poly sialylated glycoproteins NCAM[MeSH Terms] OR E-Cadherin downregulated via polysialylated NCAM[MeSH Terms] OR E-cadherin mediated cell-cell interaction[MeSH Terms] OR influenza virus's hemagglutinin [MeSH Terms] OR NDV virus's hemagglutinin neuraminidase [MeSH Terms] OR Activation of natural killer cells by Newcastle OR Virus cleaves glycosidic bond sialic acid residues OR Virus targeted cell membrane sialic acids[MeSH Terms] OR lectins contain carbohydrate affinity sites[MeSH Terms] OR membrane cell lectins possess affinities to neighbouring glycosylated proteins[MeSH Terms] OR Lectin glycan possess relatively weak affinity but their strong interactions depend sum protein[MeSH Terms] OR mammals lectins function facilitate cell-cell interactions result signal transduction[MeSH Terms] OR lectins triggered immune effector cell activation[MeSH Terms] OR lectins can be potent toxins[MeSH Terms] OR mammalian mammals' lectins differentiated into classes based various amino acid sequences[MeSH Terms] OR mammals lectins/sialylated glycoproteins interaction initiate biological processes[MeSH Terms] OR calcium dependent protein domains[MeSH Terms] OR C type lectins have common 120 amino acids, which bind glycosylated proteins[MeSH Terms] OR calcium ion linked a mannose residue to lectins Man-NAc OR mannose residue co-partners binding depends calcium[MeSH Terms] OR Selectins epithelial/lymphocytic/platelet cells have higher affinity interact carbohydrate, facilitate immune-system lymphocyte-infiltrating[MeSH Terms] OR selectins preferentially possess ability bind sulphated/ fucosialylated glycans[MeSH Terms] OR transmembrane proto-oncogene tyrosine-protein kinase encoded c-ros oncogene[MeSH Terms] OR discovered oncogene product ROS in avian sarcoma RNA virus UR2 [MeSH Terms] OR ROS1 human anaplastic lymphoma kinases structure has similarity ALK/LTK[MeSH Terms] OR macrophages transmembrane DC-SIGN bind pathogens including bacteria/fungi/viruses mannose carbohydrates[MeSH Terms] OR polysialylation B-cell receptors including CD45/IgM/GD1/ganglioside1/GT1b isolated from gram-negative bacteria Neisseria meningitidis/ Campylobacter/ Pseudomonas aeruginosa/E.coli K1/ gram-positive bacteria Streptococcus[MeSH Terms] OR pathogenic microorganisms bacteria/viruses/parasites contain sialic acids, which utilize corresponding cell surface lectins and their ligands attach infect respective cells[MeSH Terms] OR Sialic acid key component glycoconjugates sialyltransferases glycoproteins/glycolipids[MeSH Terms] OR Gram-negative bacteria pyruvate Lactate via aldolase enzyme synthesize sialic acids[MeSH Terms] OR Proteins bind carbohydrates termed lectins[MeSH Terms] OR plants, animals, viruses, bacteria express Lectins[MeSH Terms] OR Siglec1 sialoadhesin lacks immunoreceptor tyrosine-based inhibitory motif[MeSH Terms] OR sialoadhesin a positive regulator immune system, targeted sialylated bacteria, viruses envelope´s /pathogens sialoglycans [MeSH Terms] OR cancer anaplastic lymphoma kinase / pathogenic cancer[MeSH Terms] OR C-type lectin DC-SIGN able bind strong ICAM3 (CD50) /DC-SIGNR[MeSH Terms] OR DC-SIGN bind weakly polysialylated NCAM[MeSH Terms] OR DC-SIGN/ NCAM1 interactions alters intra/extracellular signal transduction activating immune system against cancer cells/ pathogens including bacterially/virally [MeSH Terms] OR unsialylated growth factor receptor clustered via

galectins exert intrinsic interaction capacity/signaling[MeSH Terms] OR imbalances between sialic acid residues /their carrier lectin-proteins /lectin-like bindings partners ligand ultimately promote resistance tumor cell and pathogens[MeSH Terms] OR pathogens parasitic/ bacterial/viral escaped innate/adaptive immune system via higher sialic acid expression[MeSH Terms] OR sialic acid based hyper reactivity associated autoimmune/neuronal diseases[MeSH Terms] OR tumor cells OR pathogens immune escape via strong inhibitory signal via sialic acid/Lectins binding[MeSH Terms] OR highlighting key immune function against pathogens/ cancer via siglec/sialic acid binding[MeSH Terms] OR siglecs 14-16 play positive/ negative immune regulation role[MeSH Terms] OR tumor cells re-express polysialylated glycoproteins analogous bacteria expressed polysialic acid[MeSH Terms] OR higher sialylation bacteria/ tumor cells become resistance apposite immune system or cancer therapeutic[MeSH Terms] OR (DC-SIGN recognized high-mannose-containing virus envelope glycoproteins including HIV/Lassa virus/ Ebola virus/ Hepatitis C virus[MeSH Terms] OR hepatitis C viruses/HIV binds dendritic cells DC-SIGN leads T-cells infection[MeSH Terms] OR DC-SIGN /NCAM1 interaction induced target lysis via natural killer cells[MeSH Terms] OR polysialylated CD56dim NK cells increased HIV infected cell-Lysis via anti-DC-SIGN antibody[MeSH Terms] OR DC-SIGN /NCAM1 interaction decreased via surface dendritic cells trans adhesion molecules neuropilin 2/ NCAM repulsion[MeSH Terms] OR dendritic cells expressed NRP-2 via higher ST8Sia IV expression[MeSH Terms] OR activated adaptive immune effector cells express polysialylated adhesion molecule[MeSH Terms])) AND (NRP2 OR NRP1 OR NCAM1 OR NCAM 2 OR PSA OR SynCAM1 OR CADM1 OR polysialic acid OR alpha-2,8 sialic acid OR alpha-2,8 sialic acid OR polysialic acid OR Plasticity OR Cell Migration OR ST8Sia OR cell adhesion glycoproteins OR poly sialylation OR polysialyltransferase OR N-Acetylneuraminic Acid OR over sialylation OR hyper sialylation OR Sialyl LewisX OR sialylated Glycan OR lactoferrin OR transferrin OR Fucosylated CD44 OR Neuraminidases OR virus/ bacteria interacts proteoglycans OR adhesion molecules L1-CAM OR E-cadherin OR Integrins)))
100345 articles, 450 duplicates, 225 hits, after removal of duplicate hits: 58 hits

[a)]**:** The study by Fossella et al. focuses on BB-10901, a humanized monoclonal antibody that selectively binds CD56. CD56 is an alternative name for NCAM, which is poly-sialylated. Multiple mechanisms underlie its antitumor activity. The connection to this article is reduction of electrostatic repulsion. BB-10901 binds to polysialylated CD56, potentially masking or neutralizing the negative charges of polySia. This can reduce electrostatic hindrance and allow closer physical contact between tumor and immune effector cells, promoting more effective immune recognition and lysis.

[b)]: Re-expression of polysialylated CD56 (NCAM) is not only important for the immune escape of tumor cells but also central for the activation of Siglecs (e.g. CD33/Siglec-3 recognition and signaling). This suppressive signal is instrumental in neuronal diseases, eg. Alzheimer’s disease. In the current article, we focus on cancer, a subsequent publication will detail the role of CD56 polysialylation on various neuronal diseases caused by inflammation. However, we have selected the literature regarding neuronal diseases here to obtain an unbiased overview.
